# Subtypes of minimal residual disease and outcome for stage II colon cancer treated by surgery alone

**DOI:** 10.3332/ecancer.2020.1119

**Published:** 2020-10-08

**Authors:** Nigel P Murray, Socrates Aedo, Ricardo Villalon, Vidal Albarran, Shenda Orrego, Eghon Guzman

**Affiliations:** 1 Servicio de Medicina, Hospital de Carabineros de Chile, Simón Bolívar 2200, Ñuñoa, Santiago 8370179, Chile; 2 Facultad de Medicina, Universidad Finis Terrae, Av. Pedro de Valdivia 1509, Providencia, Santiago 7501015, Chile; 3 Facultad de Medicina, Universidad Finis Terrae, Av. Pedro de Valdivia 1509, Providencia, Santiago 7501015, Chile; 4 Servicio de Coloproctologia, Hospital de Carabineros de Chile, Simón Bolívar 2200, Ñuñoa, Santiago 8370179, Chile; 5 Faculty of Medicine, University Mayor, San Pio X 2422, Providencia, Santiago 7601003, Chile

**Keywords:** colon cancer, circulating tumour cells, micrometastasis, minimal residual disease, prognosis

## Abstract

**Introduction:**

Twenty-five percent of stage II colon cancer (CC) patients relapse within 5 years due to minimal residual disease (MRD) not eliminated by surgery. We hypothesise that subtypes of MRD, defined by circulating tumour cells (CTCs) and bone marrow micrometastasis (mM), have different types and kinetics of relapse.

**Methods and patients:**

One month after surgery, blood and bone marrow samples were taken to detect CTCs and mM using immunocytochemistry with anti-carcinoembryonic antigen (CEA). Follow-up was for up to 5 years or relapse. Disease-free survival curves using Kaplan–Meier (DFS) and restricted mean disease-free survival times (RMST) were calculated for three prognostic groups: A: MRD (−), B: mM (+) CTC (−) MRD and C: CTC (+) MRD.

**Results:**

One hundred and eighty-one patients (82 men) have participated, mean age was 68 years and median follow-up was 4.04 years (A (N = 105), B (N = 36) and C (N = 40)). For the whole cohort of 5 years, DFS was 70%, median DFS has not reached (Groups A: 98%, B: 63% and C: 7%) and median DFS for Groups A and B have not reached. RMST for the whole cohort of 4.1 years, Group A was 4.9 years, B was 4.1 years and C was 1.7 years. Serum CEA was significantly higher in Group C. No significant differences for sex, age or high-risk adverse prognostic factors between groups were detected.

**Conclusions:**

MRD subtypes define relapse patterns and may be useful to define the risk of relapse in stage II CC patients, in which patients may benefit or not from additional therapy and warrants further studies with a larger number of patients.

## Introduction

Colon cancer represents 6.1% of all cancers and is the third most common cause of cancer mortality [1], with surgical resection being the only curative treatment option for stage I–III colon cancer [2]. Stage II colon cancer is a heterogeneous disease, and up to 25% of patients treated by surgery alone will develop metastasis [3]. Patients are classified as being at low or high risk of relapse based on the histopathological findings in the surgical specimen. Patients having one or more of the following characteristics are classified as high risk: stage pT4, poorly differentiated tumour, intestinal perforation, lymphovascular/perineural invasion and less than 12 lymph nodes examined and positive surgical margins [4–6]. Overall survival rates vary significantly, from 66.7% in stage IIA cancer to 45.7% in stage IIC cancer [7]. However, the pathological findings may not reflect the biological characteristics of different subpopulations of cancer cells present in the primary tumour. The development of metastasis is the result of occult tumour dissemination prior to surgery; tumour cells that remain after curative surgery are termed as a minimal residual disease (MRD).

We hypothesise that there are at least two subtypes of MRD. Circulating tumour cells (CTCs) detected after curative surgery are associated with decreased disease-free survival (DFS) and overall survival [8]. Tumour cells have been detected in 30% of bone marrow samples and are associated with a worse prognosis even though bony metastasis is infrequent [9]. We hypothesise that patients with CTCs detected after curative surgery are at high risk for early relapse, patients with tumour cells present only in bone marrow samples (that is CTC negative) are at risk of late relapse and those patients negative for both subtypes of MRD have an excellent prognosis. This hypothesis was supported by a recent study in stage III colon cancer patients, where the subtypes of MRD post-chemotherapy were associated with the risk and time to relapse [10].

We present a prospective observational study of stage II colon cancer patients treated with surgery, the presence of MRD post-surgery and its association with known risk factors for relapse, with DFS and time to treatment failure.

## Patients and methods

A prospective observational single-centre study of consecutive patients referred for the evaluation of minimal residual disease between January 2007 and December 2014, and those who were eligible with the following inclusion criteria: pathological Stage II colon cancer, negative surgical margins and negative CT scan of the thorax, abdomen and pelvis for metastasis.

For each patient, after giving written informed consent, the following were recorded: age, sex, date of surgical treatment, depth of primary tumour invasion (T) and nodal infiltration (N) according to the TNM classification [2]. Lympho-vascular and peri-neural infiltration in the primary tumour was recorded as present or absent, and tumour differentiation was registered as well, moderate or poor. The serum carcinoembryonic antigen (CEA) taken 1 month after surgery was registered.

### CTC detection

One month after surgery, 8 mL of blood samples were collected into tubes containing EDTA (Becton-Vacutainer®, USA).

Mononuclear cells were obtained using differential gel centrifugation with Histopaque-1077® (Sigma-Aldrich, USA) and re-suspended in 100 μL of autologous plasma, and 25 μL of aliquots were used to prepare four slides (silanized, DAKO, USA), air-dried and fixed using a solution of 70% ethanol, 5% formaldehyde and 25% phosphate-buffered saline with a pH of 7.4 (DAKO, USA).

CTCs were detected using monoclonal anti-CEA clone 11-7 (DAKO, USA) and identified using an alkaline phosphatase–anti-alkaline phosphatase-based system (LSAB2, DAKO, USA) with neofuschin as the chromogen. Positive samples underwent a second process using anti-CD45 clone 2B11 + PD7/26 (DAKO, USA) and identified using a peroxidase-based system (LSAB2, DAKO, USA) with DAB (3,3ʹdiaminobenzidine tetrachloride) as the chromogen.

**Figure 1.**
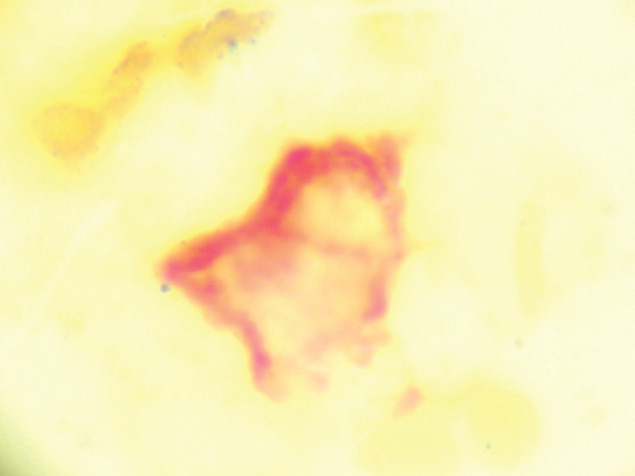
Circulating tumour cells, expressing carcinoembryonic antigen (red) and negative for membrane CD45.

**Figure 2.**
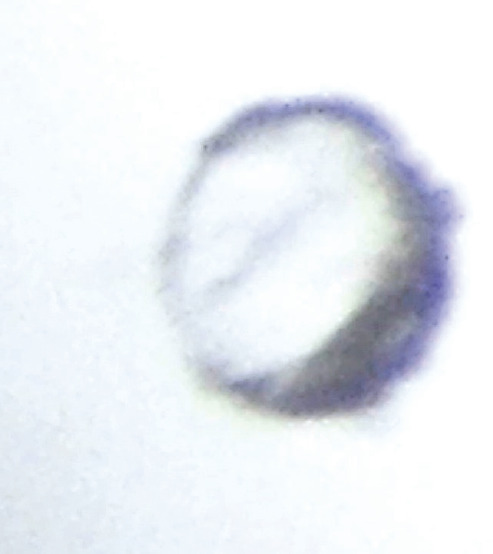
Leucocyte, negative for carcinoembryonic antigen (red) and positive for membrane CD45 (brown).

A CTC was defined according to the criteria of ISHAGE [12], as a nucleated cell expressing CEA but not CD45. A positive test was defined as the detection of at least one cell/8 mL of venous blood (Figures 1 and 2).

### Bone marrow micro-metastasis detection

**Figure 3.**
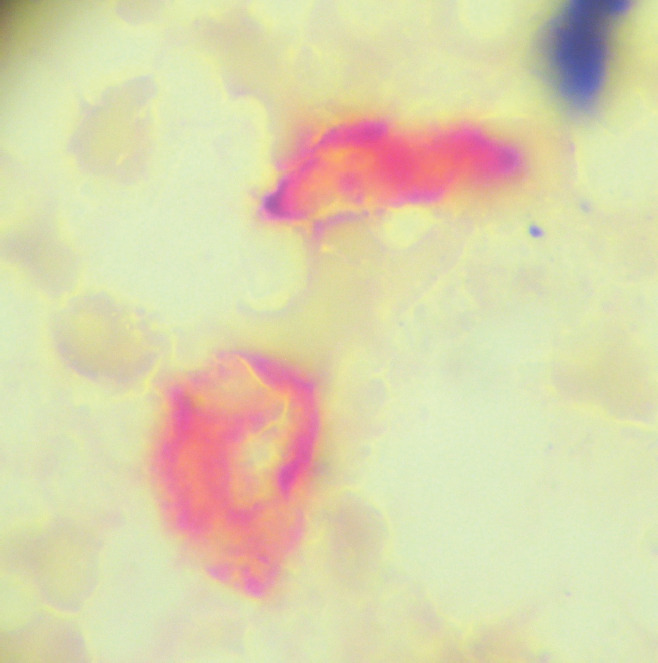
Bone marrow micrometastasis, cells expressing carcinoembryonic antigen (red) and negative for membrane CD45.

**Figure 4.**
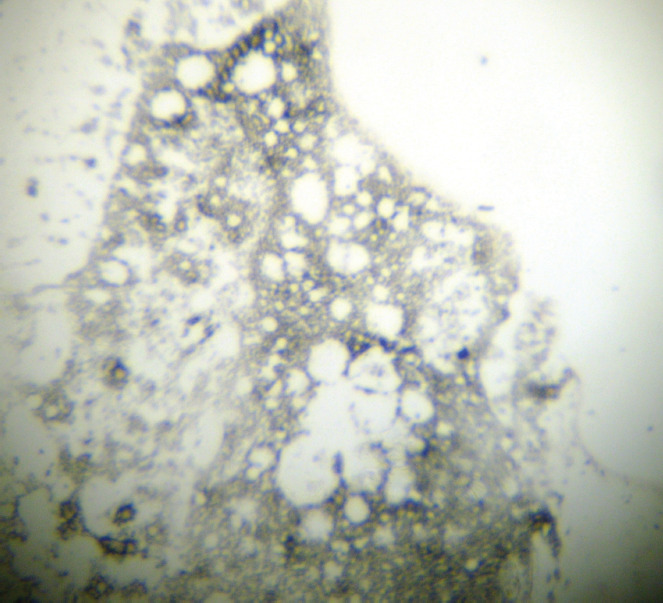
Bone marrow negative for micrometastasis (red).

At the time of CTC sampling, a bone marrow biopsy was taken from the iliac crest and used to prepare four ‘touch preps’ using silanized slides (DAKO, USA). All four slides were processed as described for CTCs, and a micrometastasis was defined as cells staining positive for CEA and negative for CD45 (Figures 3 and 4).

The patients were divided into three MRD subgroups: Group A—negative for both CTCs and micrometastasis patients, Group B—CTC negative and micrometastasis positive and Group C—CTC positive with or without bone marrow micrometastasis detected.

### Follow-up

Patients were followed up for 3 months for the first 2 years, then 6 months until 5 years. Relapse was defined as a new lesion detected on CT scanning of the thorax, abdomen or pelvis. Patients were censored at the time of relapse or after 5 years of disease-free progression; the date of the CT scan detecting relapse was used to define the time to treatment failure.

### Study end point

The primary study end point was the presence of relapse, and the secondary end point was the restricted mean time to relapse.

### Statistical analysis

The programme Stata (Stata/SE 14.0 for Windows, StataCorp LP, 20159) was used, describing quantitative and ordinate variables with measurements of central tendency (mean and median) and dispersion using the interquartile range (IQR) and standard deviation (SD). The nominal dichotomous variables were described as proportions with their respective confidence intervals.

The MRD prognostic groups were compared for age, sex, primary tumour differentiation, lymphovascular infiltration, perineural infiltration and serum CEA presurgery. The Kruskal–Wallis test was used to test whether samples originated from the same distribution, and Pearson's Chi-squared test was used to compare frequencies between MRD subgroups. A *p*-value <0.05 was taken to signify statistical significance, and all tests were two-tailed.

A nonparametric survival analysis was performed at 3 and 5 years of follow-up to determine the DFS (Kaplan–Meier) and median DFS of the whole cohort and by MRD subgroups. A nonparametric comparison (log-rank test) for DFS was performed between the MRD subgroups (Groups A, B and C).

Multivariable survival analysis was carried out using a flexible parametric survival model (FP model) to determine the hazard ratios for 5 years of follow-up [13]. The FP model is a regression method, in which the dependent variable is the survival for the studied outcome [13]. The Harrell’s-C discrimination index was used to compare predicted and observed patient outcomes. Serum CEA levels taken 1 month after surgery were compared with the number of CTCs detected, both with respect to a normal or elevated CEA and the median and range of CEA for groups 0 CTCs, 1–2 CTCs detected/sample and ≥3 CTCs detected/sample.

### Ethical considerations

The study was approved by the local ethics committee and fully complied with the Declaration of Helsinki and Chilean law on patient’s rights. All patients provided written informed consent.

## Results

One hundred and eighty-one patients were enrolled between January 2007 and December 2014, with a median follow-up of 4.0 years (IQR 2.7 years; range 0.6–5.0 years). The median age was 68 years (IQR 16 years) with a median serum CEA of 3.13 mg/mL (IQR 2.97 mg/mL), and 82 (46%) were male.

One hundred and five subjects (58%) were both CTC and micrometastasis negative (Group A), 36 subjects (20%) were CTC negative and micrometastasis positive (Group B) and, finally, 40 subjects (22%) were CTC positive, independent of the presence or absence of micrometastasis (Group C).

The comparison between groups for known clinicopathological parameters is shown in Table 1. There were no differences between groups with respect to age or sex, but the serum CEA was significantly higher in Group C. The frequency of T3 tumours was significantly higher, and lymphovascular/perineural infiltration was significantly lower in Group A, without a significant difference between Groups B and C, except that vascular infiltration was significantly higher in Group C. Well-differentiated tumours were more frequent in Group A, whereas poorly differentiated tumours in Group C. 43 (41%) Group A patients had at least one high-risk factor present, 29 (81%) in Group B and 38 (95%) in Group C (*p* < 0.05). The frequency of relapses significantly increased from Group A to Group C: Group A—2(2%), Group B—15 (41%) and Group C—33 (86%).

### Serum CEA and CTCs

One hundred and sixteen (64%) patients had a normal serum CEA postsurgery (defined as 0–5 ng/mL), and the frequency of patients with an increased serum CEA was significantly higher in CTC-positive patients (*p* < 0.001) (Table 2).

The median CEA was significantly higher in CTC-positive patients though, between patients with 1-2 CTCs/sample and ≥3 CTCs/sample, there was no significant difference, but this may be for the low sample numbers. Similarly, in patients with an increased CEA postsurgery, there was an increased frequency of CTC-positive patients. However, 56% of patients with an elevated CEA postsurgery were CTC negative with an excellent prognosis. On the contrary, only 11% of patients with a normal CEA were CTC positive.

### Disease-free survival and relapse

**Figure 5.**
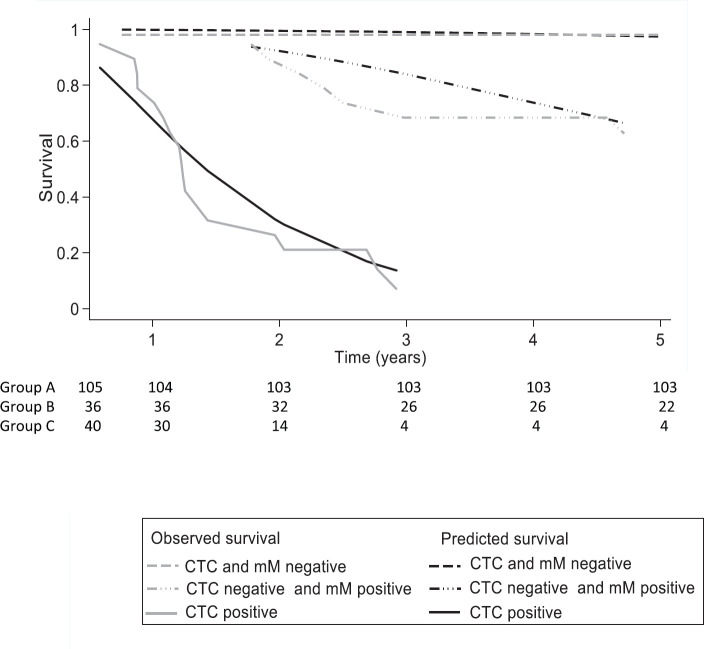
Comparing the survival prediction of the flexible parametric model (FP) versus the observed survival of Kaplan–Meier (KM) for treatment failure at 5 years according to the classification of criteria: circulating tumour cells (CTC) and bone micrometastasis (mM), in 181 individuals with colon cancer. CTC = Circulating tumour cell; mM = Micrometastasis; Observed survival = Kaplan–Meier survival; Predicted survival model FP = average proportion of mean survival for a given group determined on flexible parametric (FP) survival model. The FP model incorporates age (continuous variable), carcinoembryonic antigen (continuous variable), presence of CTC negative and mM positive (dummy variable) and presence of CTC positive (dummy variable) with one degree of freedom for the restricted cubic spline function used for the baseline hazard rate (DF1).

After 3 and 5 years, the Kaplan–Meier DFS for the whole cohort was 73.0% (95% CI: 62.5–81.0) and 71.0 (95% CI: 59.9–79.5), but the observed median DFS was not reached. The observed Kaplan–Meier DFS according to the classification of MRD criteria is shown in Table 3 and Figure 5.

The median observed DFS was not reached during the 5-year follow-up period in Groups A and B, and for Group C, it was 15 months (95% CI: 12–23 months). Group A patients had a DFS of 98% at 3 and 5 years even though 41% had factors considered to be high risk for relapse. Group C had a DFS of 7% at 3 and 5 years, and 95% were positive for factors considered to be high risk. Group B had a different pattern of relapse, and for the first 2 years, the DFS curve was similar to Group A. Thereafter, there was increasing disease relapse, 68% DFS at 3 years and 62% at 5 years. 81% had factors considered to indicate a high risk of relapse.

The FP survival model for one degree of freedom used for the baseline hazard rate (DF1) was age HR (−0.04), CEA (0.03), Group B (2.76) and Group C (5.29). The observed DFS (Kaplan–Meier) for treatment failure is concordant with the predicted DFS of the FP model (Table 2, Figure 5). The Harrell’s C discrimination index for the predicted FP model was 0.91, which is considered to be very good.

## Discussion

The current risk classification for the prognosis of stage II colon cancer is based on the pathological and morphological characteristics of the primary tumour which may not represent the biological characteristics of the tumour cells. The presence of MRD is the net result of the heterogeneous biological characteristics of the primary tumour cell population and host defence mechanisms. First, for MRD to occur, the tumour cells have to disseminate, survive in the circulation and implant and survive in distant tissues. Those tumour cells unable to complete all the steps of the metastatic process are eliminated. Of more than a million tumour cells/gram of primary tumour which can be shed daily into the circulation, only 0.1% survived to implant in distant tissues [14, 15]. Once implanted the interactions with the local microenvironment, both the stromal cells and immune system will determine the outcome, of whether the cancer cells are eliminated, enter a variable dormant period or continue to proliferate. This biological heterogeneity of cancer is seen within and between primary tumours and different metastases in the same organ or different organ microenvironments [16].

The study identified three subgroups of patients; patients negative for MRD had an excellent DFS 98% at 5 years even though 41% of these patients had at least one factor considered to be at high risk for later relapse. Those patients with CTCs detected had a very poor prognosis, with a high risk of early relapse, and 95% had factors considered to be high risk for treatment failure. Those patients with only micrometastasis showed a relapse pattern that differed with time, and initially, relapse was similar to MRD-negative patients, but after 2 years, the relapse rate increased, with a DFS of 68% at 3 years. This suggests that the balance between tumour cell characteristics and host defence mechanisms changes with time. Genetic instability of micrometastatic tumour cells and/or changes in the immune system, such as differences in the ratio between cytotoxic CD8 T-lymphocytes and regulatory CD4 T-regulatory cells, being implicated [16, 17]. The results of this observational study are consistent with reported clinical studies.

There was a statistically significant difference between the serum CEA and the number of CTCs detected in the blood sample, and an increased CEA is associated with the presence of CTCS; however, 56% of patients with an increased CEA were CTC negative. This represented 20% of the total cohort, and thus, using an elevated serum CEA as a marker to indicate adjuvant chemotherapy would result in half of the patients being over-treated.

A study of 871 stage II patients reported a median DFS of 1.9 years, that risk factors did not predict relapse and the use of chemotherapy did not improve cancer-specific survival in patients with adverse features [18–20].

The results suggest that patients with adverse risk factors may be negative for MRD, and the use of chemotherapy in these patients would not be beneficial, exposing patients to the side effects of chemotherapy. On the contrary, patients positive for CTCs possibly could benefit from the use of chemotherapy. Notwithstanding the response of MRD to chemotherapy is heterogeneous and the subtype of MRD does not predict response, approximately 40% of patients changed the subtype of MRD after FOLFOX chemotherapy [10].

Future developments in the use of CTCs and cell-free DNA (cfDNA) are in course and go beyond the simple enumeration or presence/absence as a method of monitoring patients or predicting prognosis. It has been reported that the *in vitro* culture of CTCs could be used in a cytotoxic assay to monitor drug response [21]. The molecular characterisation of CTCs to assess *KRAS*, *BRAF* and *PIK3CA* status has been achieved, which showed discordance of their status between the primary tumour and CTCs [22]. Using matched CTC and cfDNA, there was a concordance of 78.2% for *KRAS,* 73.9% for *BRAF* and 91.3% for *PIK3CA* mutations [23]. This could be used as a guide for treatment as *KRAS* mutations are frequent drivers of acquired resistance to cetuximab, and the emergence of these *KRAS* mutant clones can be detected months before radiographic progression [24, 25]. These assays can be repeated, and the cfDNA profiles which change as a result of clonal evolution and selection could be used to indicate patients who may benefit from initiating or re-challenging with targeted therapy [26, 27]. Thus, both methods of the detection of MRD could be used to guide treatment decisions and assess treatment responses during the course of therapy in order to predict drug resistance and the selection of anticancer therapy.

It is important to describe the limitations of the study, first, it was a single-centre study; second, the limited number of patients who participated and as such concrete conclusions cannot be made. However, the results suggest that the different subtypes of MRD impact patient outcome and represent a different relapse risk classification.

The detection of CTCs is method dependent, and the frequency of CTCs detected in patients with localised cancer using the EpCAM (Epithelial Cell Adhesion Molecule)-based CellSearch® system has been reported to be between 5% and 25% [28] and the size-based separation method (MetaCell®) detected CTCs in 80% of stage II patients [29]. On comparing flow cytometry, CellSearch®, quantitative real-time PCR and cytomorphology, cytomorphology showed the least sensitivity and specificity in detecting CTCs, and there were no significant differences between the other three methods [30]. Comparing RT-PCR with CellSearch®, CTCs were detected in 75% versus 20%, respectively, and in only 14% using gene mutation analysis [31]. The method would not detect CEA-negative CTCs and bone marrow micrometastasis but has the advantage of being relatively cheap, and although it is 10–100 times less sensitive than RT-PCR-based methods, it could be implemented in the routine laboratory of a general hospital. We suggest that sensitivity may not be a limiting factor; the important question is clinical utility. As yet, independent of the detection method used, there is no established lower limit of clinically significant detection to predict future relapse. Detecting every cancer cell may not be important; patients postallogeneic bone marrow transplantation for leukaemia may have a very small number of leukaemic cells detected by RT-PCR in bone marrow samples but remain in remission for many years. Furthermore, these leukaemia cells may survive for prolonged periods before being eradicated by host defences [32]. Thus, ultrasensitive methods to detect tumour cells may over-estimate clinically significant minimal residual disease in patients with solid tumours.

## Conclusions

The study suggests that treatment outcome in stage II colon cancer is dependent on the subtype of MRD; its detection identifies patients who may or may not benefit from additional treatment. These results need to be confirmed with a larger number of patients.

## Conflicts of interest

Dr Murray has received consultancy fees from Viatar CTC Solutions, Boston, USA.

## Authors’ contributions

Concept: NPM; design: NPM and SA; supervision: NPM, RV and VA; resources: NPM; materials: NPM, SO, RV and VA; data collection and/or processing: SA, RV, VA, EG and SO; analysis and/or interpretation: NPM, SA, RV, VA, EG and SO; literature search: SO and EG; writing manuscript: NPM, SA and EG; critical review: RV, VA, ER and SO and final approval: all

## Funding

The work was supported by a Western Metropolitan Health Authority Research Grant. The Funding source did not participate in the design of the study, the recruitment of patients, data collection, analysis or interpretation.

## Figures and Tables

**Table 1.** Clinicopathological findings according to classification by criteria: circulating tumour cells and bone marrow micrometastasis, in 181 individuals with colon cancer, who were followed up for up to 5 years.

Characteristics	Group A MRD negative	Group B CTC (-) mM (+)	Group C CTC (+)	*p*-Value
N° Patients	105	36	40	
Age (years) median:IQR Sex (male) CEA (ng/mL) Median:IQR	71:14 44 (42%) 2.85:2.43	68:15 16 (44%) 2.91:2.74	68:22 22 (55%) 7.84:6.92	0.64^a^ 0.48^b^ 0.005^c^
**Tumour size** T3 T4	92 13	21 15	19 21	<0.0001^b^ B versus C *p* = 0.5
**Infiltration** Lymphatic Vascular Perineural	18/105 (17%) 16/105 (15%) 5/105 (5%)	14/36 (39%) 19/36 (53%) 4/36 (11%)	23/40 (58%) 35/40 (88%) 9/40 (235)	<0.002^b^ B versus C *p* = 0.33^b^ <0.0001^b^ B versus C 0.04^b^ *p* = 0.03^b^ B vs C *p* = 0.5^b^
**Differentiation** Well Moderate Poorly	70/105 (67%) 23/105 (22%) 12/105 (11%)	17/36 (47%) 13/36 (36%) 6/36 (17%)	5/40 (12%) 24/40 (60%) 11/40 (28%)	<0.0001^b^ B versus C <0.005^b^
**N° Factors High Risk** 0 1 2 3 4	62 (59%) 17 (16%) 16 (15%) 10 (9%) 0 (0%)	7 (19%) 10 (27%) 12 (33%) 5 (13%) 2 (5%)	2 (5%) 15 (37%) 10 (25%) 6 (15%) 7 (17%)	<0.001^b^ B versus C *p* = 0.16
**Relapse** N (%)	2 (2%)	15 (41%)	33 (86%)	<0.001^d^

IQR = Interquartile range; CTC = Circulating tumour cell; mM = Micrometastasis

a Kruskal–Wallis test

b Pearson's Chi-squared test

c *post hoc* analysis showed significant differences between Groups A and C

d Pearson's Chi-square test with the Marascuilo procedure for *post hoc* analysis showed significant differences between Group A versus B, Group A versus C and Group B versus C

**Table 2.** Association between serum CEA and CTCs.

	CTC negative	1-2 CTCs/sample	≥ 3 CTCs/sample
CEA normal *N* = 116 (0–5 ng/mL)	103 (89%)	11 (10%)	2 (1%)
CEA increased *N* = 65 (>5 ng/mL)	37 (56%)	16 (24%)	12 (18%) *p* < 0.001^a^
Median CEA (ng/mL) (IQR)	2.91 (2.04–3.41)	6.71 (2.77–15.3)	13.4 (6.59–24.8) 0 versus 1–2 *p* < 0.001^b^ 0 versus ≥ 3 *p* < 0.001^b^ 1–2 versus ≥ 3 *p* = 0.49^b^

CEA = Serum carcinoembryonic antigen; CTC = Circulating tumour cell

a Chi-squared,

b Mann–Whitney test

**Table 3.** Comparing observed survival (Kaplan-Meier) versus predicted (Model FP) for treatment failure at 3 and 5 years according to criteria: circulating tumour cells and bone micrometastasis, in 181 individuals with colon cancer.

Variable predictor		3 years		5 years	
		Survival observed^a^ % (95% CI)	Survival predicted^b^	Survival observed^a^ % (95% CI)	Survival predicted^b^
Prognostic group	A CTC and mM negative *n* = 105	98.08 (87.12–99.73)	99.07	98.08 (87.12–99.73)	97.47
	B CTC negative and mM positive *n* = 36	68.42 (42.79–84.39)	83.97	62.72 (37.25–80.22)	63.42
	C CTC positive *n* = 40	7.02 (0.51–26.23)	12.69	7.02 (0.51–26.23)	1.75

%: percentage; CI = Confidence interval; CTC = Circulating tumour cell; mM = Micrometastasis

a Observed survival = Kaplan–Meier Survival

b Predicted survival model FP = average proportion of mean survival for a given group determined on flexible parametric (FP) survival model. The FP model incorporates age (continuous variable), carcinoembryonic antigen (continuous variable), presence of CTC negative and mM positive (dummy variable) and presence of CTC positive (dummy variable) with one degree of freedom for the restricted cubic spline function used for the baseline hazard rate (DF1)
